# How Structural Compensation Facilitates Health Care for the Homeless. A Comparative View on Four European Union Member States

**DOI:** 10.3390/ijerph17239114

**Published:** 2020-12-06

**Authors:** Ursula Trummer, Sonja Novak-Zezula, Mariola Chrzanowska, Christos Michalakelis, Roido Mitoula, Adam Rybka, Snejana Sulima, Monika Zielińska-Sitkiewicz

**Affiliations:** 1 Center for Health and Migration, 1090 Vienna, Austria; sonja.novak-zezula@c-hm.com; 2 Department of Econometrics and Statistics, Warsaw University of Life Sciences, 02-787 Warsaw, Poland; mariola_chrzanowska@sggw.edu.pl (M.C.); monika_zielinska_sitkiewicz@sggw.edu.pl (M.Z.-S.); 3 Department of Informatics and Telematics, Harokopio University of Athens, 17778 Tavros, Athens, Greece; michalak@hua.gr; 4 School of Environment Geography and Applied Economics, Harokopio University of Athens, 17676 Kallithea, Attiki, Greece; mitoula@hua.gr; 5 Department of Town Planning and Architecture, Faculty of Civil and Environmental Engineering and Architecture, Rzeszow University of Technology, 35-959 Rzeszów, Poland; akbyr@prz.edu.pl; 6 Faculty of Law, Alexandru Ioan Cuza University, 700506 Iasi, Romania; snejanasulima@yahoo.com

**Keywords:** health care, homeless, structural compensation, vulnerable groups, inequality in health

## Abstract

There is robust evidence that homelessness and the associated life conditions of a homeless person may cause and exacerbate a wide range of health problems, while healthcare for the homeless is simultaneously limited in accessibility, availability, and appropriateness. This article investigates legal frameworks of health care provision, existing knowledge on numbers of homeless to be considered, and current means of health care provision for four EU countries with different economic and public health background: Austria, Greece, Poland, and Romania. National experts investigated the respective regulations and practices in place with desk research. The results show differences in national frameworks of inclusion into health care provision and knowledge on the number of people experiencing homelessness, but high similarity when it comes to main actors of actual health care provision for homeless populations. In all included countries, despite their differences in economic investments and universality of access to public health systems, it is mainly NGOs providing health care to those experiencing homelessness. This phenomenon fits into conceptual frameworks developed around service provision for vulnerable population groups, wherein it has been described as “structural compensation,” meaning that NGOs compensate a structural inappropriateness that can be observed within public health systems.

## 1. Introduction

There is a robust body of evidence concerning the manifold nexus of health and homelessness, with ill health being a cause and an outcome of homelessness.

Early studies indicate that homeless individuals are disproportionately at risk for a wide range of illnesses and injuries compared to the general population. The condition of homelessness and the exigencies of life of a homeless person may cause and exacerbate a wide range of health problems [1]. It is also noted that health problems themselves may contribute to a person becoming or remaining homeless, such as in the cases of mental illness and substance use, particularly in the absence of treatment facilities and supportive housing arrangements [1]. A study aiming to clarify what comes first, homelessness or substance abuse, and using datasets from two services in Melbourne, supplemented with in-depth interviews, shows that 43% of the sample had substance abuse problems, with two-thirds of these having developed the problems after becoming homeless [2]. Furthermore, the state of being homeless makes treatment and management of most illnesses more difficult, even if services are available [1]. It is pointed out that, in both the US and Europe, homeless people typically attend the emergency department more often than non-homeless people [3]. 

There is also evidence showing that the health needs of homeless people are widely unmet for a variety of reasons. In the US context, lacking health insurance is a major obstacle to receiving adequate care [4]; within the context of the European Union and its associated welfare state models, this variable is comparatively less decisive. Programs focused on high-risk groups, such as individuals leaving prisons and psychiatric hospitals, as well as the introduction of national and state-wide plans that target homeless people are named as likely to improve outcomes in terms of the physical and mental health of homeless people [3]. Still, a secondary analysis of cross-sectional data from a sample of 2437 homeless adults in Spain (83.8% male) on self-rated health showed that—besides other factors such as sleeping difficulties or having a disability/impairment—having a health card was a significant factor associated with perceived good health [5].

The negative ramifications of homelessness on both physical and mental health have also been shown to have a serious effect on life expectancy. A population-based cross-sectional study for the UK, concerning causes of mortality amongst individuals who are homeless, identified that their median age of death was 51.6 compared to 71.5 for a random sample of residents living in socially deprived neighborhoods [6]. Higher rates of premature mortality amongst the homeless, relative to the general population, have been associated with suicide and unintentional injuries, and an increased prevalence of a range of infectious diseases, mental disorders, and substance misuse [3]. 

Despite the poor health statuses of homeless individuals, the services available to this population are commonly described as being insufficient, and limited in their accessibility, availability and appropriateness [7]. Studies also refer to a medicalization process among homeless services and point to specific examples of services denying homeless people’s access entry unless they conform to a medicalized model [8]. 

This article aims to provide information on health services for homeless people in four EU countries: Austria, Greece, Poland and Romania. The choice of these four included countries is the outcome of an initiative led by the Austrian authors of this article within the COST Action CA15218—measuring homelessness in Europe [9]. In this COST action, experts from 32 European countries cooperate to bring together expertise and knowledge with regard to measuring homelessness, to tackling measurement challenges and to creating a common European framework on measuring homelessness. In the framework of an expert meeting in March 2019 in Bratislava, the Austrian COST Action members called for experts to participate in a survey on national regulations concerning access to health care for homeless people. Experts from four countries declared their interest in the topic of health care for the homeless. In a dedicated workshop within the framework of the Bratislava meeting, researchers discussed and agreed on guiding questions for their respective search and analysis on national level. 

Guiding questions are:

1. What are the legal and structural frameworks for health care provision in the respective countries?
2. Which—if any—reliable counts or robust estimates of the number of people homeless are available, and is there additional information—e.g., numbers of specifically vulnerable groups, such as migrants, women, children, elderlies?
3. What kind of services providing health care for homeless populations are available in the respective countries in terms of care provided (general/specialized) and organizational types (governmental/non-governmental)?

## 2. Materials and Methods

A national-level country overview was prepared by national experts in the field of homelessness, for each country under study.

The choice of experts was done through the self-selection of national representatives to the COST action on “Measuring homelessness in Europe”.

National experts collected information along the guiding questions for their respective countries. Results were collected by the Austrian experts and an overview on expert inputs from all four countries was sent back to national experts for feedback. Experts being native speakers in the language of majority within their respective country of study ensured that legal regulations, statistical data, and reports available in the local language could be used as a source and interpreted correctly.

Desk research included (1) a search for national laws and regulations regarding health care for the homeless, (2) a search on numbers of homeless people and their characteristics, such as age, sex, and citizenship in national and European/OECD statistics, (3) an internet search for organizations providing health care for the homeless with the keywords health care/health care provision combined with homeless, uninsured, vulnerable groups, (4) a consultation of the identified organizations’ websites, and (5) a search for national, regional or local reports on the practice of health care provision for the homeless. 

Cross-country differences concerning the availability of data and priority setting of country experts did put limitations on synthesizing results. Authors decided to allow different prioritizations within the guiding questions of search and in the presentation of national findings. The following section on country-level results reflects this diversity in priority setting.

## 3. Results

### 3.1. Austria

#### 3.1.1. Legal and structural frameworks for health care provision

The Austrian health care system is insurance-based and provides access to a broad range of services for people holding health insurance. The system is described as being close to universal in coverage [10], with a reported 99.9% of the population holding health insurance [11]. All people who are employed, self-employed, retired, unemployed or co-insured as dependents are health insured. Since the introduction of a needs-based basic income in 2010, recipients of these social benefits are also provided with health insurance and have unrestricted access to public health services. All of these regulations apply to Austrian citizens and migrants with a regular status.

#### 3.1.2. Numbers on Homelessness

Official national statistics give a total number of 21,567 registered homeless people for 2017. Among them are 13,926, holding a so-called “Hauptwohnsitzbestätigung” (main domicile confirmation). According to §19a MeldeG 1991, the Hauptwohnsitzbestätigung may be issued by registration offices provided that the homeless individual can verify that his or her center of life has been in the respective municipality for at least one month and can name a contact point that he or she visits regularly. Included within the population of homeless individuals, registered at an appropriate registration office and holding a Hauptwohnsitzbestätigung, are rough sleepers, people in emergency accommodation, people living in non-conventional dwellings due to a lack of housing, or homeless people living temporarily in conventional housing with family and friends due to lack of housing. Another 8688 are registered as residing in services for the homeless [12]. 

A report on inclusion indicators for Austria in 2018, analyses the socio-demographic structure of 22,741 registered homeless people [13]. While 26.9% were homeless for the entirety of the year, 73.1% experienced episodes of homelessness. In total, 86% were registered in municipalities with more than 100.000 inhabitants. This includes the capital city of Vienna, where 57% of all registered homeless individuals are living. The share of men among the registered homeless is 69.2%. However, it is stated that the applied methods of counting the homeless might underestimate the number of women affected, due to the specific forms of homelessness women experience. They are assumed to be more greatly affected by hidden homelessness, staying with family and friends due to their homelessness, and are not registering with authorities to the same extent as men. This statement is substantiated by the Viennese social services, which highlight that 51% of eviction prevention measures are used by women, while only 30% of the available homeless services are used by women [14]. The age structure shows that about two-thirds of those experiencing homelessness are between 25 and 64 years old; 6.7% are aged 65+ compared to 18.8% of the overall population. The absolute numbers of male and female homeless under the age of 18 are similar, as they are almost exclusively registered in institutions for homeless youth. Among the registered homeless, 44.1% are foreign citizens.

#### 3.1.3. Health Care Provision for the Homeless

According to the legal framework described above, homeless Austrian citizens and migrants with a regular status can be included in the health insurance system and can accordingly access public health services. However, a number of homeless individuals are unable or otherwise do not want to contact authorities or comply with necessary regulations for obtaining health insurance. Additionally, there is an unknown number of undocumented migrants who are not allowed to obtain health insurance, as they do not hold a regular residence permit [15]. 

Health care for uninsured homeless people in Austria, as well as those who are insured but experience other barriers in accessing public health care, is mainly provided by non-governmental organizations (NGOs). For primary health care, there are a small number of NGOs that are dedicated to providing primary care to everyone in Austria without insurance [16]. These NGOs are financed by public health and social welfare institutions and by donations. Volunteer work from health professionals also plays an important role in economic terms. A secondary analysis of a European database for Austria for 2014 shows that four NGOs that provide health care for uninsured people manage to mobilize the volunteer work of health professionals with an equivalent value of around Euro 587.000 per year [17]. 

These NGOs are located in Austria’s biggest cities: Vienna, Austria’s capital, Graz/Styria, Linz/Upper Austria, Salzburg/Salzburg, and Innsbruck/Tyrolia. They organize care in the context of different settings, including primary care centers, mobile units, or in the framework of hospital outpatient departments.

##### Primary Care Centers

Primary care centers are Neunerhaus and Ambermed in Vienna, the Marienambulanz in Graz and Medcar(e) in Innsbruck. The Marienambulanz and Medcar(e) additionally organize mobile units.

Neunerhaus, a social organization located in Vienna, runs a primary care center for homeless people, and further provides dental care and additional health services through mobile doctors who see patients in Neunerhaus shelters. The NGO aims at assuring a self-determined life, characterized by dignity, to homeless people and those living in poverty, through the provision of health care, housing and counselling. The organization prioritizes empowerment, with the goal of working towards sustainable improvement to the living situation of homeless and socially disadvantaged individuals and engages against the exclusion of homeless people [18].

AmberMed is an NGO comprised of a volunteer team of health professionals and interpreters, run by the Diakonie Flüchtlingsdienst, which provides free medical care, including medicine (in cooperation with the Red Cross), to uninsured people who are permanently in Austria. AmberMed works to reduce health inequality and promote patient integration into the Austrian health system. Although the service is not per definition dedicated to the homeless, they are among the clients [19].

Marienambulanz, an NGO operated by Caritas in Graz, provides low-threshold primary care, including the provision of medication (in cooperation with the Red Cross), counselling and social work services to uninsured individuals and those otherwise unable to access public health services due to barriers such as language, poverty and addiction. In addition, the Marienambulanz organizes a mobile bus that visits public places and shelters where homeless individuals live, in order to provide care to those who cannot visit the Marienambulanz surgery [20].

Medcar(e) is a service providing social and basic medical care to the homeless in Innsbruck. Run by Caritas, Medcar(e) operates both a permanent practice and a mobile unit, which visits different public places in the community once a week [21].

##### Mobile Units 

Mobile units providing basic health care to homeless patients are the Louisebus in Vienna, Help-Mobil in Linz, and Virgilbus in Salzburg.

Louisebus, a mobile unit operated in Vienna by Caritas, provides primary care to homeless people who are unable to visit public health services for different reasons. A team of doctors and volunteers see patients five days a week at seven defined locations in Vienna. Louisebus defines itself as a low-threshold parallel structure that compensates for insufficiencies in the public health care system by providing services adapted to the needs of this vulnerable clientele [22].

Virgilbus is a mobile unit run by the Red Cross, which provides emergency medical care to homeless individuals within the municipality of Salzburg, many of whom are emergency travelers from Southeast Europe. Supported by volunteer physicians, paramedics, and interpreters, the service operates one evening a week [23].

Help-Mobil is a joint service by Caritas, the Arbeitersamariterbundes OÖ, the Kongregation der Barmherzigen Schwestern Linz, the Red Cross Linz and the Lazarus-Ordens Hilfsdienst Oberösterreich. Help-Mobil is a bus that goes to several stops in Linz, two evenings per week, to provide basic medical care and medicine free-of-charge to the homeless. The service also offers counselling and provides homeless individuals with necessity items, such as sleeping bags, clothing and food, as needed [24].

##### Hospital Services 

In Vienna, there is one hospital explicitly offering health care to vulnerable patients without asking for an ID or health insurance, called the Barmherzige Brüder Hospital. The outpatient unit is accessible for uninsured patients, including those who are homeless. In the event that inpatient care is deemed necessary, uninsured patients are transferred to the hospital ward following approval by an interprofessional board. Both outpatient and inpatient health care provided to uninsured patients are financed through collected donations [25]. The Barmherzige Brüder Hospital is a confessional hospital under public law in Vienna.

### 3.2. Greece 

#### 3.2.1. Legal and Structural Frameworks for Health Care Provision

According to Law 4368/2016 and JMD A3 (c)/DG/51/25132/4-4-2016, uninsured and vulnerable social groups in Greece, including those who are homeless, are granted the right of free access to all public health infrastructure for the provision of nursing and medical care. The health coverage guaranteed by this new framework is comprehensive and includes nursing and diagnostic and pharmaceutical coverage for vulnerable populations. Accordingly, the homeless are entitled to the same services offered by public health care infrastructure to insured individuals, including [26]:

- Free access to primary and secondary public health infrastructure, mental health units, rehabilitation facilities, and university hospitals.
- All nursing and diagnostic services at no charge.
- Scheduled surgeries free of charge.
- Preventative health services (e.g., vaccinations) free of charge.
- Free dental care.
- Free maternity care and benefits from public hospitals.
- Medication supplies by private and public pharmacies—a large part of the uninsured population is entitled to medication free of charge.
- Physiotherapy, speech therapy, occupational therapy, psychotherapy and special treatment actions.
- Administration of medical aids and consumables.
- Access to emergency departments.

#### 3.2.2. Numbers on Homelessness

According to the FEANTSA Country Fiche Greece (update 2018), a first census for homeless people in seven main Greek cities in May 2018, showed 691 individuals living in the streets, 516 living in shelters and 438 in apartments for the homeless [27]. In regard to personal characteristics, the results indicate that the profile of the homeless in Greece seems to be primarily Greek men, aged 18–44. The country fiche quotes a study published in 2015, which estimates 17,720 rough sleepers in the prefecture of Attica and up to 500,000 persons experiencing various forms of homelessness as described by ETHOS typology [27]. Among specific groups affected by homelessness, listed in this country fiche are unaccompanied minors, victims of trafficking, and refugees. In general, it is estimated that there has been a rapid increase in the number of individuals experiencing invisible homelessness as a result of financial, economic and social crises [27].

#### 3.2.3. Health Care Provision for the Homeless

##### Access to Local Government Offices in Greece

Along with the National Health System, local authorities have created social clinics for disadvantaged citizens across numerous municipalities in Greece. These offices are staffed and equipped by regional and municipal professionals and operate on a voluntary basis. Clinics have various specialties, including general medicine, pathology, cardiology, pediatrics, gynecology and ophthalmology. Patients are also offered vaccination and prevention services, treatment for conditions where no hospitalization is required, prescription medication, and inpatient care. However, many of these services lack systematic follow-up of patients, particularly for those with chronic illnesses. Accordingly, there are some questions concerning service spectrum, quality and safety. The shortage of follow-up care for the poor and uninsured is evident in their vaccination coverage, which remains fragmented and uncoordinated despite commendable efforts by voluntary and social welfare bodies. These conditions raise alarming and pessimistic concerns [28].

Non-Governmental Organizations and other actions.

The provision of primary care to the homeless is often handled by NGOs alongside other fragmented actions. In this case, services rely on volunteering, which stems from a sense of compassion for the fellow human being. Following are two typical examples.

##### PRAKIS

The NGO PRAKSIS designs and implements humanitarian action programs in Greece. In particular, the organization aims to provide free social and medical services in the fields of treatment, prevention, education, and health infrastructure. The organization further aims to promote solidarity and volunteering, through the development of programs that combat both the social and economic exclusion of vulnerable people and groups. Working alongside official government agencies, local authorities, additional NGOs, and the broader Greek society, PRAKSIS strives to create networks to effectively meet the needs of its target groups. Basic health-related services provided by PRAKSIS include:

- Direct and free medical care.
- Basic hygiene services (showers, hygiene kits, clothes, etc.).
- Psychological support.

PRAKSIS operates throughout Greece, primarily in Attica, Central Macedonia, the Island of Lesbos and Patras. The NGO also operates mobile units, which travel to the islands of the North Aegean, the Dodecanese, Northern Greece, Thessaloniki and Promachonas, as well as according to any additional needs that arise [29]. 

##### EMFASIS

Through its daily work and activities in Athens, the EMFASIS Foundation understands the needs of vulnerable social groups and continually strives to address these needs promptly and effectively. The organization is active in the health sector through the provision of free medical examinations, which are available to those experiencing homelessness, as well as otherwise socially disadvantaged individuals lacking medical insurance. The purpose of this organization is to help those deprived of their right to health care and to support these individuals by improving their quality of life and living conditions through the provision of health cards.

The free medical exam packages covered by EMFASIS health cards are selected and tailored to the basic needs of men, women and children and include, inter alia, general, gynecological, urological and pediatric examinations, which upon completion are evaluated by a general practitioner.

The EMFASIS team, made up of trained scientists (psychologists, sociologists and social workers), receives requests for health cards from uninsured individuals lacking access to doctors or the means to cover their medical expenses [30].

In general, issues relating to homelessness in Greece are being addressed with fragmented efforts. The homeless are treated as disadvantaged, thereby making it difficult to estimate their numbers, as they are part of a broader set of low-income social groups that have access to the public health system and local government social clinics.

### 3.3. Poland 

#### 3.3.1. Legal and Structural Frameworks for Health Care Provision

In the Constitution of the Republic of Poland (Article 68), it is written that everyone has the right to health protection, and citizens, regardless of their financial situation, should have equal access to health care services financed from public funds. The provisions of Polish law do not distinguish specific rules for the provision of healthcare services for the homeless.

In the case of insured persons, their treatment is financed from the resources derived from health insurance contributions at the disposal of the National Health Fund. Healthcare services provided to other beneficiaries entitled to free health services, but who are not insured, are financed from state budgetary funds allocated to the Minister of Health or Interior and Administration or Justice, in the form of subsidies, as referred to in paragraph 97, Section 8 of the Act of 27 August 2004 on health care benefits financed from public funds. Therefore, in the case of uninsured individuals, such as those experiencing homelessness, these benefits are financed from the state budget on the basis of subsidies and the public payer (National Health Fund) is only an intermediary in payments for the services available to those who are uninsured.

Most often, homeless individuals may acquire entitlement to healthcare benefits following the receipt of a positive decision from the commune head (mayor or president) of the relevant commune, issued on the basis of paragraph 54, Section 1 of the Act of 27 August 2004 on health care benefits financed from public funds. The right to healthcare services based on this decision is valid for a period of 90 days and gives the legal basis for reimbursement of such person’s medical expenses by the National Health Fund.

A homeless person may also be covered by health insurance as part of the Homelessness Leaving Programme, pursuant to the provision of paragraph 66 Section 1 point 29 of the Act of 27 August 2004 on health care benefits financed from public funds. In practice, uptake of these programs is minimal, and few homeless people acquire the right to health care services financed from public funds. Many individuals experiencing homelessness still encounter difficulties in accessing health services due to bureaucratic requirements, and health care institutions often exhibit a marked reluctance to apply the relevant legislation.

Homeless people in Poland are mainly helped by NGOs. For years, these organizations have been experiencing problems due to rules restricting their activity. The main problem is that legal regulations create a system that is not adapted to the needs of individual people. Everyone who actively helps the homeless acknowledges that individual history is of paramount importance; it gives an understanding of the underlying problems and is also the key to support. Therefore, help should be personalized. Currently, however, the opposite situation exists, wherein the Act on social assistance and other regulations governing the work of homeless organizations creates a network of systems and programs to which the homeless individual must adapt in order to receive care. Unfortunately, in most cases this is not possible.

In addition, homeless seniors form the largest subpopulation amongst those experiencing homelessness in Poland, and it is this group who is most likely to experience chronic homelessness as well as the biggest health problems. Consequently, proposed solutions for expanding medical assistance and social care should be adjusted to the needs of homeless seniors [31].

Acts on social assistance specify that financial responsibility for homeless assistance is borne by the commune under which a homeless individual last held a permanent address. Often, such a person is no longer associated with this commune. This legislation, therefore, creates many problems for homeless individuals and for communes. For example, the funeral of a homeless person needs to be addressed and payed. However, when communes pass on responsibility between themselves, the waiting time for a burial is sometimes several months. Another issue is the difficulty of shelter access for dependent homeless individuals, including the elderly in need of permanent assistance or those who have a disability. There is also trouble in financing the NGOs’ shelter services by the municipalities. The amendment to the Act on Social Assistance from 2016—the “Municipal Standard of Exiting Homelessness” framework—has been largely criticized for creating such loopholes [32]. 

#### 3.3.2. Estimate the Scale of Homelessness in Poland

Since 2009, the Polish Ministry of Labor and Social Policy has been estimating the scale of homelessness in Poland by conducting coordinated research across the country on a single night, in order to avoid counting the same people twice or on several occasions. The first data collection was taken at night on 15 December 2009, but because of the surprisingly good weather conditions at the time, it had to be repeated on 26 January 2010, when the temperature had dropped significantly. The aim of the census was to determine the number of overnight stays for homeless people in the voivodeships, meaning basic regions for the application of regional policies, of Poland. A national survey of the number of homeless people is conducted cyclically—every second winter. Table 1 shows the results of homeless counting in Poland.

The exact number of homeless people living in Polish territory is unknown. Researchers estimate the number of homeless Poles to be in the range of 30,000 to 100,000. Such a large difference in the reported data is related to the lack of reliable assessments of the number of homeless people living in institutions (institutions, night shelters) and other places (allotments, gardens, garages, and railway and bus stations).

Estimations from non-governmental institutions indicate that there are more homeless people in Poland than those counted by national surveys. It is assumed that approximately one-third of homeless people took advantage of the accommodations provided by institutions for the homeless, despite severe weather conditions on the nights that surveys were conducted. Discrepancies in the statistics occur due to the fact that some homeless people use help from several sources, which increases the total number of people covered. Furthermore, accurate estimation of the number of people experiencing homelessness is difficult because not all of these individuals use available forms of assistance. In addition, homeless individuals move around the country much more often than other groups.

#### 3.3.3. Health Care Provision for the Homeless

##### Access to Government and Non-government Health Care in Poland

Theoretically, homeless individuals should receive health care in hospitals in Poland, but unfortunately this help is not always provided in the right way. Admission to hospital and ensuing inpatient care for a person experiencing homelessness is hampered by the related procedure of obtaining health insurance. Applicable regulations formally create such a possibility, but it is preceded by a very complicated process, which is not adapted to the conditions of homelessness and situations of people experiencing such a state. The procedure for obtaining health insurance requires the completion of documents. It does not fulfill its tasks for homeless patients who, by the nature of things, do not have a permanent address. There are additional problems with providing care after hospitalization. Homeless people often go to the street, which significantly hinders or sometimes prevents the healing and treatment process. In this case, there is a lack of coordination between healthcare and social services.

Hospitals have the biggest problem in the event of lost consciousness. They must first apply to the court for a probation officer, which is necessary to develop case law, obtain permanent benefit and start procedures. Court procedures are protracted, with even a slight formal mistake lengthening the process, as the patient waits in hospital for the procedure to finish. Usually such a person needs to obtain an ID card and go through the procedure of determining the degree of their disability. Together, this lasts for just over two months. Only then can homeless individuals apply for permanent allowance to the Municipal Social Welfare Center and for insurance, and finally for placement (e.g., in a nursing home).

The experiences of institutions that help homeless people on a daily basis, most of whom are NGOs, corroborate this problem. The imperative role played by NGOs in providing care for homeless populations emphasizes the shortcomings of state instruments, because NGOs, by definition, grow where fields poorly handled by the state care have appeared. In other words, NGOs fill in the gap between insufficient government activities and refugees needs.

People who have to use homeless facilities are in very diverse situations and therefore have different needs. In Poland, practitioners’ experiences show that many homeless people have serious health problems, such as malnutrition, anemia, diabetes, sores, frostbite, pneumonia, tuberculosis, mental illness or disability. In many cases, it is impossible to start the difficult work of getting out of homelessness without solving the underlying health conditions of these people. Some NGOs try to adapt their services to emerging needs through specialization and division of labor.

Based on the research and reports of Polish experts, Julia Wygańska and Adriana Porowska, the following forms of non-governmental health care for the homeless can be distinguished in Poland [38]:

- Medical assistance provided in institutions receiving emergency homeless people in response to an immediate need (e.g., night shelters, shelters). This includes nursing and basic medical assistance as well as provision of medicines and dressings.
- Inpatient facilities, such as hospitals and isolators, by definition intended “for patients”, sometimes called specialized facilities (Warsaw), to which homeless persons are admitted in a state of health that prevents them from staying in a “normal” institution for the homeless. Help from a nurse, internist, and sometimes a psychiatrist is provided. Medicines and dressings are also available.
- Homes for the elderly, the sick and the disabled. They provide help from nurses and specialist doctors as well as access to medicines and dressings.
- Medical facilities, health clinics or other outpatient health care facilities—for example, private doctors’ offices that provide basic and sometimes specialist medical assistance, medicines and dressings. This group includes mainly primary health care facilities.

In virtually every major Polish city (e.g., Warsaw, Cracow and others), in which the problem of homelessness occurs, institutions specializing in the care of homeless populations have emerged. NGOs that help homeless people can finance their own activities using a variety of sources. These include national public funding (from special assistance programs), personal or institutional philanthropy, 1% tax deduction, support from other NGOs and foreign public funding (e.g., European funding).

##### The Most Important Non-Governmental Organizations 

An NGO worth noting is The Doctors of Hope Association, which operates in Krakow, Warsaw and Wroclaw. The Association runs clinics and pharmacies. Clinics for people experiencing homelessness provide consultations, issue medicines, provide referral for specialist examinations, carry out minor surgical and nursing procedures, and try to help patients return to life in society. Homeless individuals can count on the help of volunteer doctors with specializations such as internist, cardiologist, surgeon, dermatologist, laryngologist, psychiatrist, pulmonologist and dentist. Charity pharmacies of the Association issue medicines free of charge, especially for people experiencing homelessness or those living on the edge of poverty, in addition to preparing drug sets for nursing homes, shelters and for foreign missions [39].

Caritas is also an organization actively working for the homeless in Poland. From 2015, Caritas Poland and the Warsaw City Guard have run the initiative "A bit of warmth for a homeless person." Twice a week, from November to the end of February, the street patrols delivered hot soup and bread, blankets, warm clothes, and other necessities to homeless people who were trying to survive the wintertime. In 2017, Caritas Poland and the Warsaw City Guard started one more project, namely the "Medical Street Patrol," which aims to provide ambulatory medical help to the homeless who do not live in shelters. The action is supported by the Doctors of Hope Association. The support offered to the homeless includes physical exams, medical consultations, help on the spot, referral to a specialist, transfer to either a hospital or a Doctors of Hope clinic, as well as providing motivation and information on how to get out of homelessness [40].

The St. Brother Albert Aid Society has been active since 1981, and it was the first non-governmental organization in Poland that started to help the homeless. It has 2900 members organized in 62 Circles (branches). Each Circle tries to establish a hostel, soup kitchen or other kind of help for the poor and homeless, including basic medical help. The society accomplishes its aims through:

- Running hostels, night shelters, permanent residence homes, soup kitchens, bath houses, day rooms and clubs.
- Social work, legal help, psychological help and priesthood.

Every year, the St. Brother Albert Aid Society organizes Christmas Eve suppers, Easter breakfasts and summer camps. The distribution of food, clothes, appliances, household detergents and medicines are also an important aspect of their charity. They cooperate with local and central governments, the Catholic Church, and non-governmental organizations, and run information activities and train staff and volunteers [41].

The Monar-Markot Association offers help to homeless people in dozens of different types of facilities across Poland. The association runs shelters, soup kitchens, stationary homes and crisis intervention facilities for those experiencing homelessness and poverty, and offers specialist legal, psychological and social help. There are stationary therapeutic programs for homeless individuals with a drug or alcohol consumption problem. In addition, the Monar-Markot runs its medical clinics and hospitals for homeless people without health insurance. Special medical help and therapeutic support is offered for homeless individuals with different problems. The association has its own homelessness leaving program and also runs shelters for single mothers with children, for the elderly, for those leaving prison and for evicted families.

##### Other Action

The Citizens’ Rights Ombudsman in Poland is also strongly interested in access to health care for people experiencing a homelessness crisis and often intervenes in situations where a hospital refuses to help a homeless person. The Ombudsman’s institution has an Expert Committee on Combating Homelessness, which reports to relevant ministries about the need for systemic change and the creation of an efficient system that combines medical and social care.

### 3.4. Romania 

#### 3.4.1. Legal and Structural Frameworks for Health Care Provision

According to Romanian legislation (Law 292/2011), a homeless person is “someone represented in a social category formed by a single people or families who, because of singular or cumulated reasons (social, medical, financial, economic or legal) or because of force majeure, lives on the streets or with friends or acquaintances and is unable to sustain a rented house or is threatened with eviction, or lives in institutions or prisons and is due to be released within two months and lacks a domicile or residence”.

To obtain medical assistance in Romania, the patient must be contributing to the health system; otherwise, people are entitled to 72 h of emergency treatment only. In practice, most Romanians contribute to the health system through formal work contracts; however, many low-skill jobs can only be found in the informal sector. Accordingly, these employees cannot be registered to a family doctor and are excluded from the health care system, with the exception of emergency treatment. Furthermore, although health services are free of charge to those who are insured, the reality is that medical procedures are unlikely without informal payments, which can be very expensive.

Social Health Insurance (SHI) is compulsory but covers only around 86% of the population. This figure may be misleading, however, as a very significant number of Romanians (between 3 and 4 million) are working abroad but are still counted as being in Romania. Insured individuals are entitled to a comprehensive benefits package, while the uninsured are entitled to a minimum benefits package, which covers only life-threatening emergencies, epidemic-prone/infectious diseases and care during pregnancy. Romania’s health system is characterized by low funding and inefficient use of public resources, with the lowest spending per capita and share of its GDP in the EU. There is a lack of universal coverage, although the non-covered population does have access to a minimum package of benefits. There are also inequities with regard to access to services between the rural and urban populations and for vulnerable populations. Recent efforts include the creation of community care centers to improve access, including for the Roma population. 

Health problems are strongly associated with the pathways to homelessness [42]; however, there is scarce available evidence on the effectiveness of healthcare services for homeless people in Romania. On the other hand, life on the streets is associated with serious health problems, but there is a very limited access to social services providing medical assistance [43]. Chronic diseases and mental illnesses are more common among the homeless compared to the general population. There are some national policies to address the phenomenon of homelessness, but the current state initiatives are limited to providing social support, access to emergency healthcare, and emergency and temporary housing. At the same time, several NGOs are providing medical care, education (for minors), psychosocial support and, sometimes, housing. However, their efforts are limited in terms of coverage and are highly dependent on the availability of funding, especially from international donors [44]. 

#### 3.4.2. Numbers on Homelessness

According to the FEANTSA Country Fiche Romania (update 2017), very few data are available on homelessness in Romania. It is stated that, among the “marginalised persons” registered with the authorities in 2011, about 41,000 did not own or rent a place to live and about 162,000 lived in inadequate conditions [45]. 

#### 3.4.3. Health Care Provision for the Homeless

At the local level, there are public social and medical assistance services in big cities, which offer some primary medical assistance to homeless people in addition to other services (temporary hosting, psychological assistance, etc.). There are also some private institutions offering complex assistance to homeless people, including primary medical assistance (e.g., Organizaţia Umanitară Concordia (Bucarest); Asociaţia Samusocial din România (Bucarest); Asociaţia de Asistență Socială Umanitară Creştină Ora International (Harghita); Asociaţia Caritas Alba Iulia-Asistenţă Medicală şi Socială (Harghita); Fundația Nădejdea Copiilor din România (Podu Iloaiei); Asociaţia Smiles (Sinmartin); Asociaţia Româno-Germană Alsterdorf (Oradea); Asociația pentru Servicii Sociale Comunitare Pro Humanitas (Auseu). Many of these private centers are backed by the Church or international NGOs. 

A study by Save the Children on street children reveals an important discrepancy in the help provided by state authorities and NGOs for accessing medical assistance for homeless children and youth [46]. While 60% of the respondents indicated several NGOs (ARAS, Caritas, Concordia, Parada, Salvati Copiii, Samuscal), only 5% of them mentioned the assistance of the state (especially the social assistance services and children’s protection services) in their problems.

According to the recent legislation, adopted in 2019 (Annex no. 4 to the Instruction of the Ministry of Labour and Social Justice no. 29/2019), in temporary residences, homeless people have access to the medical care of a nurse. The professionals working in these residences should assist homeless people to register in the healthcare social assistance system and help facilitate access to medical services in other specialized institutions for health, such as family health institutions or hospitals. 

The legislation adopted in Romania, in 2019, concerning the social protection of vulnerable people, should also improve the situation of the homeless. In accordance with the recent legal provisions, these individuals should be assisted in gaining access to medical care for their immediate needs as well as continuous care for long-term health concerns through registration within the healthcare system.

In order to start shaping tailored policies, there is an urgent need for the assessment of homelessness at a national level, at least in major cities. Comprehensive information based on accurate measurements would give a faithful picture of the dimensions of this phenomenon and of the differences between regions. The systems built upon these data will allow for further definition of adapted approaches to homeless health issues. The constant monitoring of homelessness requires collaboration between public institutions and specialized NGOs. 

The support for vulnerable people should include health assistance moving gradually from emergency services to long-term integration programs. Services dedicated to the homeless have to be integrated in a comprehensive package including multiple elements: from their identification and registry to their access to healthcare, allowances, pensions, shelters and housing, education, and employment opportunities. Medical assistance has to be part of a minimum support provided by the state’s coordinated system that would associate the NGOs providing care for homeless people.

## 4. Discussion

Homeless people experienced obstacles accessing health in all four countries with NGOs playing a major role in health care provision and operating in parallel to public health systems, despite a considerable heterogeneity of countries under study in regard to economic and health system parameters. 

To illustrate heterogeneity, three indicators that describe public investments in health care and the sensitivity of health care systems for economic inequalities can be of use: (i) numbers on health care expenditure relative to the GDP, (ii) the percentage of government and compulsory insurance coverage in health spending in relation to out of pocket payments, and (iii) numbers on unmet needs for health care. 

Regarding health care expenditure relative to the GDP, Eurostat data from 2017 ranks Austria as number 4 among the EU member states with a share of 10.4%, Greece in rank 15 with 8.0% and Poland in rank 23 with 6.5%. Romania is the member state with the lowest share of health care expenditure relative to the GDP with 5.2%, at rank 27, respectively [47]. 

According to health spending in 2018, in Austria 74% of total health spending is covered by government and compulsory insurance, while 19% of health spending is out-of-pocket payment. For both Greece and Poland, the percentage of government and compulsory insurance coverage is lower, with 61% for Greece and 69% for Poland, and the percentage of out-of-pocket-payments is higher (Greece 35%; Poland 23%). Expenditure by voluntary health insurance and other sources bring the differences to 100% [48]. For Romania, 2018 OECD data are not available. A report by the European Observatory on Health Systems and Policies for 2014 presents the share of health care spending as 80% public sector health expenditure and 20% private expenditure. However, it is pointed out by the authors that the extent of private expenditure may be underestimated as a result of unofficial payments [49]. 

Regarding unmet needs for health care, an indicator used to assess equity in health care denotes Austria on the one hand and Greece, Poland and Romania on the other, as two ends of the spectrum in an overview on EU countries. In Austria, 0.4% of the persons aged 16 or over felt they needed health care in the previous 12 months but did not receive it for reasons related to financial barriers, long waiting lists and transportation problems. In Greece, this value was 10.2%, in Poland 8.5%, and 6.9% for Romania [50].

These indicators display considerable differences between the four countries concerning public investments in health care, the need for out of pocket payments, and unmet health care needs. However, when it comes to addressing the needs of those experiencing homelessness, it seems to make little difference how much countries do invest in health care, whether out of pocket payments are high or low, and to what degree the general population experiences unmet health care needs. The main service providers when it comes to health care for homeless people stay the same; it is mainly NGOs that ensure health care provision for individuals experiencing homelessness. 

Furthermore, even well financed, low-threshold public health systems seem to rely on NGOs for the integration of vulnerable population groups into health care provision. This is in line with available evidence on the challenges public health systems encounter when it comes to serving the most vulnerable and hard to reach population groups who live on the edge of society [51].

Studies in the field of health care provision for another marginalized group, namely undocumented migrants, have described this phenomenon as “structural compensation” [16,52]. 

It means that NGOs set up structures to provide health care for vulnerable groups, with a considerable share of staff members working as volunteers in pursue of their ethical values. Just to give an example, the voluntary work contribution from four Austrian NGOs amounts to EUR 578,240 per year [53]. 

Such “Structural compensation” that compensates for the lack of appropriate services in the official public health system has been discussed in two rivalling aspects: (i) as fostering sustainability of deficits within the public health systems, and (ii) as a necessary element of care provision alongside the public health system fostering sustainable inclusion of hard to reach groups [16].

## 5. Conclusions

There is robust evidence on the need to provide health care to homeless people, as well as on the specific challenges related to service provision for this population from both academic discourses and organizations working in the field. Results show differences in national frameworks of inclusion into health care provision and knowledge on numbers of homeless, but high similarity when it comes to main actors of actual health care provision for those experiencing homelessness. In all included countries, despite their differences in economic investments and universality of access to public health systems, it is mainly NGOs providing health care to homeless people. This phenomenon fits into the conceptual frameworks developed around service provision for vulnerable population groups, wherein the compensation provided by NGOs for structural inappropriateness observed within public health systems is described as “structural compensation.” 

Again, the question can be raised whether it is a good solution that NGOs take over public health responsibilities in providing health care for population groups that do not fit into mainstream services, or if such “structural compensation” helps to postpone necessary adaptions of public health structures.

In all four countries under study, interesting models of service provision are in place and can be used as models to learn from. Further studies on the role of NGOs and their specific contribution and capability to serve vulnerable population groups are recommended. 

## Figures and Tables

**Table 1:** Number of homeless in Poland.

Time	Number of Homeless	Men	Women	Children
**2010**	43,083	35,321	5673	2055
**2013**	30,712	24,522	4361	1538
**2015**	36,161	28,918	5351	1892
**2017**	33,408	27,316	4891	1201
**2019**	30,330	24,901	4437	992

Source: Ministry of Labor and Social Policy reports [33,34,35,36,37].
